# Surgical approach and insert constraint are associated with small differences in patellar tilt after robotic‐assisted total knee arthroplasty: A retrospective comparative radiographic study

**DOI:** 10.1002/jeo2.70933

**Published:** 2026-10-03

**Authors:** Clément Favroul, Mehdi Drissi Kaitouni, Elvire Servien, Cécile Batailler, Sébastien Lustig

**Affiliations:** ^1^ Orthopaedics Surgery and Sports Medicine Department, FIFA Medical Center of Excellence, Croix‐Rousse Hospital, Hospices Civils de Lyon Lyon North University Hospital Lyon France; ^2^ LIBM‐EA 7424, Interuniversity Laboratory of Biology of Mobility Claude Bernard Lyon 1 University Lyon France; ^3^ University Lyon, Claude Bernard Lyon 1 University, IFSTTAR Lyon France

**Keywords:** medial parapatellar approach, mini‐subvastus approach, patellar alignment, patellofemoral joint, robotic‐assisted total knee arthroplasty

## Abstract

**Purpose:**

Patellofemoral alignment remains important after total knee arthroplasty (TKA). Robotic‐assisted functional positioning may reduce the impact of approach‐ and implant‐related factors on the patellofemoral joint. This study evaluated the association between surgical approach and insert constraint with postoperative static patellar alignment following robotic‐assisted TKA.

**Methods:**

This retrospective comparative radiographic study included 320 primary nonresurfaced robotic‐assisted TKA performed with the MAKO system between March 2021 and December 2024. Patients were divided into four groups: mini‐subvastus condylar‐stabilised (MSV‐CS, *n* = 134), mini‐subvastus posterior‐stabilised (MSV‐PS, *n* = 63), medial parapatellar condylar‐stabilised (MPP‐CS, *n* = 75) and medial parapatellar posterior‐stabilised (MPP‐PS, *n* = 48). Standardised Merchant axial radiographs at 30° and lateral radiographs were obtained at 2 months and 1 year. Patellar tilt was the primary outcome. Secondary outcomes were qualitative patellar centreing, patellar thickness and the modified Blackburne–Peel index. Two‐way factorial analysis assessed surgical approach, insert constraint and their interaction.

**Results:**

Baseline characteristics and final intraoperative robotic alignment parameters were comparable across groups. MSV and CS implants were associated with statistically greater patellar tilt than MPP and PS implants, respectively, but the absolute differences were small, approximately 1°. No significant interaction was observed between surgical approach and insert constraint. Patellar height did not differ between groups.

**Conclusion:**

Robotic‐assisted functional positioning provided consistent static patellofemoral alignment regardless of surgical approach or insert constraint. Although statistically significant differences in patellar tilt were detected, their approximately 1° magnitude suggests a negligible radiographic effect. Their clinical significance remains unknown because clinical outcomes and dynamic patellar tracking were not assessed.

**Level of Evidence:**

Level IV.

**Abbreviations**
CS: condylar‐stabilised
MPP: medial parapatellar approach
MSV: mini‐subvastus approach
PCA: posterior condylar axis
PROMs: patient‐reported outcome measures
PS: posterior‐stabilised
SD: standard deviation
TKA: total knee arthroplasty
TS: total‐stabilised
VV: varus–valgus

## INTRODUCTION

Total knee arthroplasty (TKA) provides reliable pain relief and functional improvement in end‐stage knee osteoarthritis, but 15%–20% of patients remain dissatisfied after surgery [5]. Patellofemoral complications are among the most frequent causes of residual symptoms and may present as anterior knee pain, patellar malalignment, instability or functional limitation [20].

Patellofemoral function after TKA is influenced by multiple factors, including patellar resurfacing strategy, trochlear insert constraint, femoral component rotation, tibial component position, limb alignment, soft‐tissue balance and surgical approach [9, 15]. Deroche et al. reported no superiority of patellar resurfacing over nonresurfacing with a modern prosthesis design [8], whereas other authors have emphasised the influence of alignment strategy on patellar tracking [22]. Patellar alignment is influenced by several interdependent factors, including femoral rotation, trochlear geometry, tibial component position, limb alignment, soft‐tissue balance, patellar morphology and patellar management [10, 14].

Robotic‐assisted TKA improves the precision and reproducibility of implant positioning [7, 12]. This may be particularly relevant to the patellofemoral joint, because femoral rotational malposition and trochlear orientation are recognised contributors to patellar malalignment and abnormal patellofemoral loading [15]. Beyond accuracy alone, robotic‐assisted functional positioning allows intraoperative adjustment of component position according to patient‐specific anatomy, ligament balance and patellofemoral behaviour. Recent robotic‐assisted TKA studies have also suggested that anterior compartment restoration may influence functional outcomes, although the relationship between radiographic patellofemoral parameters and clinical results remains debated [9, 13].

Even when implant positioning is planned and executed using robotic assistance, exposure and soft‐tissue handling may still affect patellar alignment. The mini‐subvastus (MSV) approach preserves the quadriceps tendon and vastus medialis continuity [2, 21], whereas the medial parapatellar (MPP) approach provides reproducible exposure but involves proximal extension into the quadriceps tendon [2]. Insert constraint may also play a role through differences in posterior cruciate ligament management and femorotibial kinematics, even when implants share a similar trochlear geometry [14, 15].

The aim of this study was to evaluate the association of surgical approach and insert constraint with static radiographic patellar alignment after nonresurfaced robotic‐assisted TKA. The primary outcome was patellar tilt on postoperative Merchant radiographs. We hypothesised that surgical approach and insert constraint could be associated with postoperative radiographic patellar alignment, without significant interaction between both factors.

## MATERIAL AND METHODS

### Study design and population

This retrospective comparative radiographic study included consecutive patients who underwent primary robotic‐assisted TKA using the MAKO robotic‐arm‐assisted system (Stryker, Kalamazoo, MI, USA) at a single institution between March 2021 and December 2024. The study focused on nonresurfaced native patellae to isolate the influence of surgical approach and insert constraint on static patellar alignment.

During the study period, 669 robotic‐assisted TKAs were screened. Patients were excluded if the patella was resurfaced (*n* = 340, 50.8%), if a TS implant was used (*n* = 8) or if a lateral parapatellar arthrotomy was performed for valgus deformity (*n* = 1). Total‐stabilised (TS) implants were excluded because they represent a more constrained design and were generally used in a clinically different population. The resurfacing decision was based partly on preoperative anterior knee pain, patellofemoral osteoarthritis severity and surgeon preference. Because resurfacing decisions incorporated surgeon preference and surgical approach was also surgeon‐dependent, differential selection across approach groups cannot be excluded. The final cohort included 320 primary robotic‐assisted TKA from 320 unique patients with nonresurfaced patellae.

Patellar resurfacing was generally considered in the presence of preoperative anterior knee pain and/or advanced patellofemoral osteoarthritis. Native patellae were preferentially retained in patients without anterior knee pain and with absent or limited patellofemoral degeneration. Patients were divided into four groups: mini‐subvastus condylar‐stabilised (MSV‐CS) (*n* = 134), mini‐subvastus posterior‐stabilised (MSV‐PS) (*n* = 63), medial parapatellar condylar‐stabilised (MPP‐CS) (*n* = 75) and medial parapatellar posterior‐stabilised (MPP‐PS) (*n* = 48) (Figure 1).

**Figure 1:**
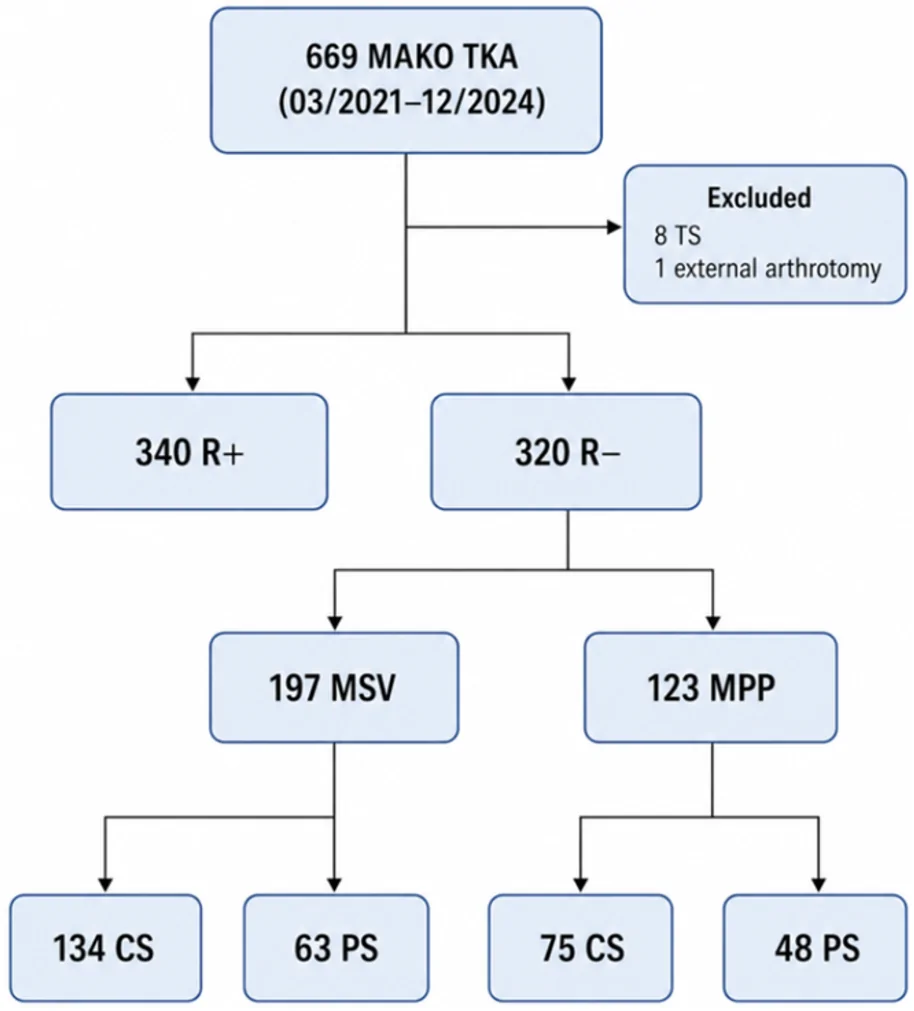
Flowchart of the study. *R* + (resurfaced TKA)/*R* − (nonresurfaced TKA). CS, condylar‐stabilised; MPP, medial parapatellar posterior; MSV, mini‐subvastus; PS, posterior‐stabilised; TKA, total knee arthroplasty; TS, total‐stabilised.

### Surgical technique and implants

All procedures were performed by three experienced knee surgeons. Two of the three surgeons used both the MSV and MPP approaches during the study period, whereas the third surgeon used only the MPP approach. Therefore, although approach selection reflected surgeon preference and was not randomised, the surgical approach was not structurally or perfectly confounded with surgeon identity, as within‐surgeon variation was present for two surgeons. Nevertheless, residual confounding by the surgeon could not be excluded. All cases were planned and performed using the MAKO system with computed tomography‐based planning.

A robotic‐assisted functional alignment strategy was used in all cases according to the principles described by Shatrov et al. [19]. Component positioning was individualised using computed tomography‐based three‐dimensional planning and intraoperative assessment of medial and lateral compartment laxity under stress. Implant position was iteratively adjusted in the coronal, sagittal and axial planes to reproduce the patient‐specific joint‐line orientation and achieve the predefined balance targets. In extension, the target residual laxity was 0 mm in both the medial and lateral compartments. In flexion, CS constructs with posterior cruciate ligament preservation were balanced towards 1.0 mm of medial and 1.5 mm of lateral residual laxity, whereas PS constructs were balanced towards 0 mm in both compartments, as in extension. Tibial varus was limited to 5°, the target posterior tibial slope was 3°, and femoral internal rotation relative to the posterior condylar axis was not accepted. Final component positions were adjusted to achieve these targets while remaining within the functional alignment boundaries. Final tibial component rotation was not routinely exported to the study database and was therefore unavailable for analysis.

All patients received components from the Triathlon total knee system (Stryker). CS constructs were used with preservation of the posterior cruciate ligament, whereas PS constructs incorporated a cam‐post mechanism. Although the femoral components belonged to the same implant family and shared identical anterior flange and trochlear geometry, the groups differed jointly in insert design, posterior cruciate ligament management and flexion‐balance targets. The CS–PS comparison should therefore be interpreted as a comparison between composite implant and balancing strategies rather than as the isolated effect of insert constraint. Construct selection was not randomised and followed surgeon preference and institutional practice.

### Radiographic evaluation

Radiographic evaluation was performed at 2 months and 1 year. Standardised Merchant axial radiographs and strict lateral radiographs were obtained at 30° of knee flexion using a goniometer. Radiographs not meeting institutional quality criteria were repeated. Preoperative Merchant radiographs were not obtained systematically. Consequently, preoperative patellar tilt, translation, morphology and patellofemoral osteoarthritis severity were unavailable for consistent analysis. The study compared postoperative measurements only and could not adjust for baseline patellofemoral characteristics.

Patellar tilt was measured on the Merchant view as the angle between the maximal transverse axis of the patella and the trochlear reference line of the femoral component. Positive values indicated lateral tilt. Patellar position was qualitatively assessed on standardised Merchant axial radiographs according to the relationship between the median patellar ridge and the prosthetic trochlear groove. The patella was classified as centred when the median patellar ridge remained engaged within the prosthetic trochlear groove, subluxated when the median ridge extended beyond the trochlear margin while partial patellofemoral engagement was maintained, and dislocated when the patella was completely disengaged from the prosthetic trochlear groove. Because dislocation was uncommon, subluxated and dislocated patellae were combined into a noncentred category for statistical analysis. Representative examples of the three categories are provided in Figure 2. Patellar thickness was measured as the maximal anteroposterior dimension of the native patella and expressed in millimetres. Patellar height was assessed using the modified Blackburne–Peel index [3]. The radiographic measurement methodology is illustrated in Figures 3, 4, 5, including patellar thickness, modified Blackburne–Peel index, patellar tilt and qualitative patellar position assessment. Positive coronal alignment values indicated valgus, and negative values indicated varus.

**Figure 2:**
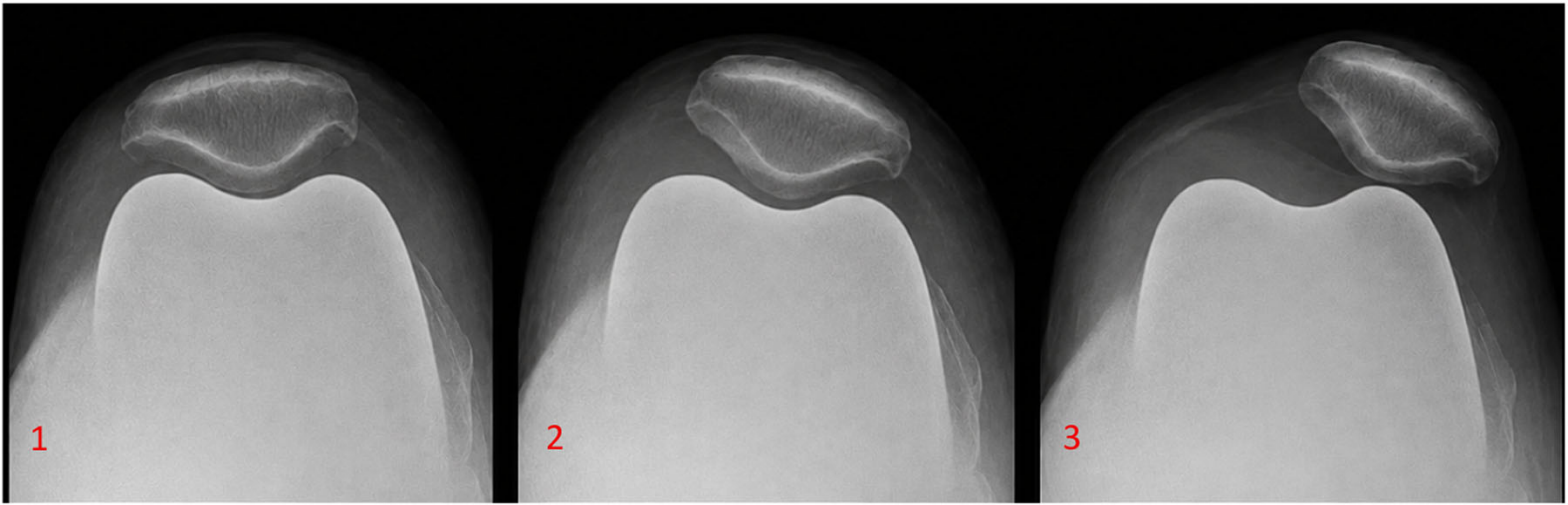
Qualitative assessment of patellar position on standardised Merchant axial radiograph. Patellar position was classified as centred (1), subluxated (2) or dislocated (3) according to the relationship between the patellar ridge and the prosthetic trochlear groove.

**Figure 3:**
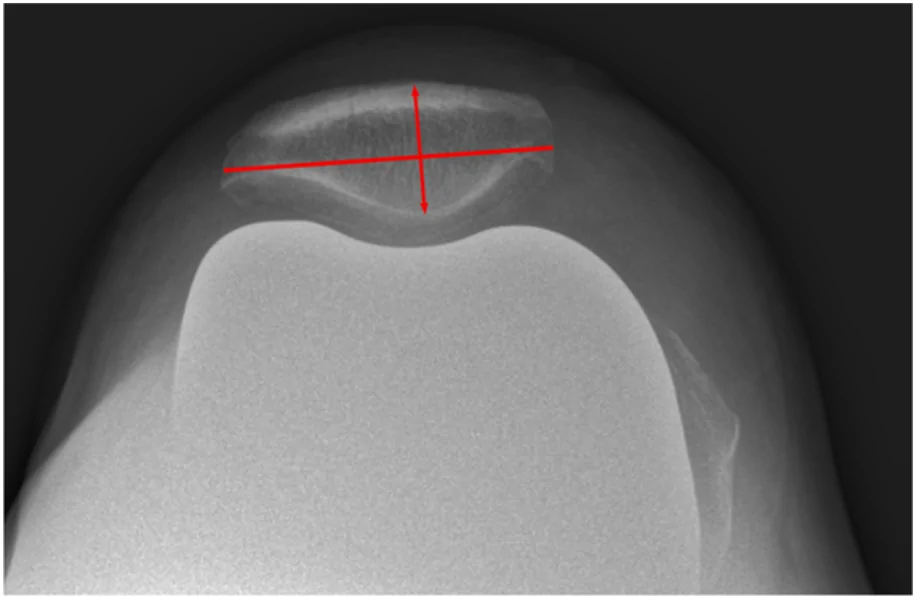
Patellar thickness measurement on standardised Merchant axial radiograph. Patellar thickness was measured as the maximal anteroposterior dimension of the native patella and expressed in millimetres.

**Figure 4:**
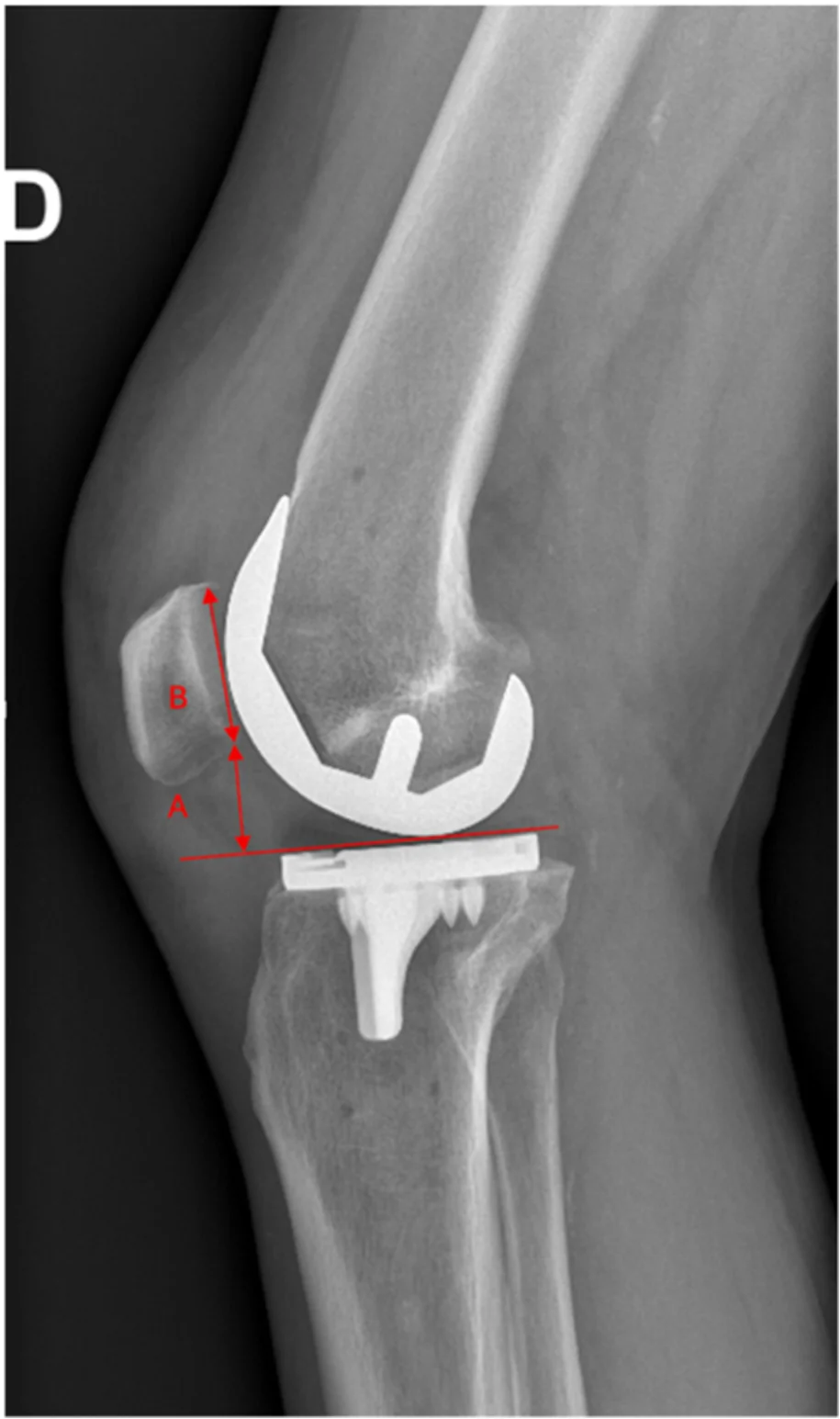
Modified Blackburne–Peel index measurement on lateral radiograph. Patellar height was assessed using the modified Blackburne–Peel index, calculated as the A/B ratio on a strict lateral radiograph.

**Figure 5:**
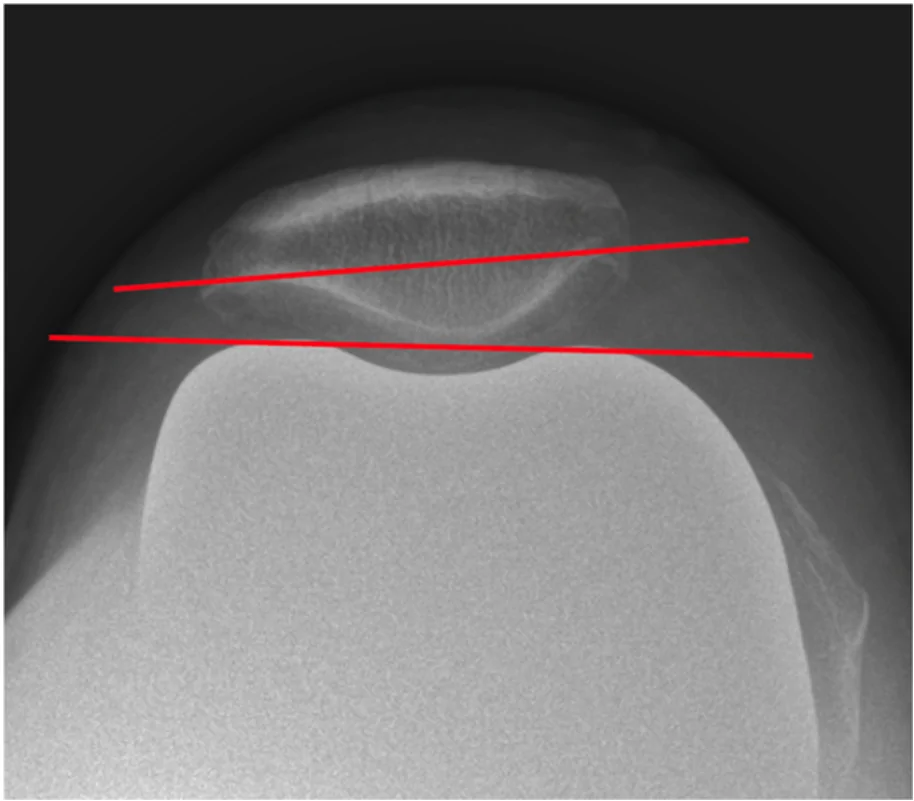
Patellar tilt measurement on standardised Merchant axial radiograph. Patellar tilt was defined as the angle between the maximal transverse axis of the patella and the trochlear reference line of the femoral component. Positive values indicated lateral tilt.

All measurements were performed by a single nonblinded observer using Centricity Universal Viewer Zero Footprint (version 6.0 SP7.0.2—GE Healthcare), which enables angle measurements with an accuracy of 0.1 degrees. To assess intraobserver reliability, a random sample of radiographs was re‐evaluated by the same observer, blinded to the initial measurements. Intraobserver reliability was assessed in a random sample of 30 knees remeasured by the same observer after a 4‐week interval, with the observer blinded to the initial measurements. A two‐way mixed‐effects, absolute‐agreement, single‐measure intraclass correlation coefficient was used. The 95% confidence interval was estimated from the reported ICC and sample size. Absolute measurement error was estimated using the residual standard deviation of the factorial model, with the standard error of measurement calculated as standard error of measurement (SEM) = standard deviation (SD) × √(1 − ICC).

### Statistical analysis

Continuous variables were expressed as mean ± SD and categorical variables as numbers and percentages. Baseline characteristics and final intraoperative robotic alignment parameters were compared between the four groups using analysis of variance or chi‐square/Fisher exact tests, as appropriate. For continuous outcomes, estimated marginal mean differences derived from the two‐way factorial model were reported with 95% confidence intervals. Standardised effect sizes were expressed as Cohen's *d* using the residual standard deviation of the factorial model.

The primary outcome was patellar tilt at 2 months and 1 year. A two‐way factorial analysis evaluated the effect of surgical approach (MSV vs. MPP), insert constraint (CS vs. PS) and the approach × implant interaction. Secondary outcomes included patellar thickness, modified Blackburne–Peel index and qualitative patellar position. Statistical significance was set at *p* < 0.05. Because this retrospective study included all eligible cases from the institutional database, no formal a priori sample‐size calculation was performed. Analyses were performed using SPSS version 26.0 (IBM Corp.).

### Ethical approval

All procedures were performed in accordance with the ethical standards of the institutional and/or national research committee, the 1964 Helsinki declaration and its later amendments, or comparable ethical standards. Data collection and analysis were carried out in accordance with MR004 Reference Methodology from the Commission Nationale de l'Informatique et des Libertés (Ref. 2229975V0) obtained on 6 May 2023. The study was registered and filed on the Health Data Hub website.

## RESULTS

### Baseline characteristics, robotic alignment and ICC

Baseline demographic characteristics and final intraoperative robotic alignment parameters were comparable across groups (Table 1). No significant differences were observed for age, weight, sex distribution, femoral coronal alignment, tibial coronal alignment or femoral rotation relative to the PCA. At 2 months and 1 year, Merchant and strict lateral radiographs were evaluable for all 320 knees. No radiograph was excluded from the final analyses, and no imputation was required.

**Table 1:** Demographic and robotic alignment characteristics of the four subgroups.

Parameter	MSV‐CS (*n* = 134)	MSV‐PS (*n* = 63)	MPP‐CS (*n* = 75)	MPP‐PS (*n* = 48)	*p*‐Value
Age (years)	73.4 ± 9.0	71.9 ± 8.4	73.5 ± 7.8	71.2 ± 7.1	0.724
Weight (kg)	79.1 ± 17.6	80.3 ± 14.3	80.2 ± 13.5	82.7 ± 14.6	0.757
BMI (kg/m^2^)	27.8 ± 4.8	28.2 ± 4.4	28.3 ± 4.2	28.4 ± 4.1	0.654
Sex (Male/Female)	65/69	32/31	37/38	14/34	0.081
Femoral VV (°)	1.21 ± 2.64	1.06 ± 1.65	0.58 ± 1.69	1.20 ± 1.69	0.079
Tibial VV (°)	−3.62 ± 1.44	−3.26 ± 1.55	−2.93 ± 1.49	−2.92 ± 1.45	0.341
Femoral rotation PCA (°)	2.82 ± 1.51	2.61 ± 1.82	2.57 ± 1.65	3.06 ± 1.74	0.119

*Note*: Values are presented as mean ± SD unless otherwise indicated.

Abbreviations: BMI, body mass index; MPP‐CS: medial parapatellar condylar‐stabilised; MPP‐PS, medial parapatellar posterior‐stabilised; MSV‐CS, mini‐subvastus condylar‐stabilised; MSV‐PS, mini‐subvastus posterior‐stabilised; PCA, posterior condylar axis; SD, standard deviation; VV, varus–valgus.

Intraobserver reliability was evaluated in 30 knees remeasured after a 4‐week interval. For patellar tilt, the ICC was 0.91 (95% CI: 0.82–0.96). Based on the residual standard deviations of the factorial models, the estimated SEM was 0.78° at 2 months and 0.71° at 1 year.

### Patellar tilt

In the factorial analysis, MSV was associated with greater lateral patellar tilt than MPP at 2 months, with an adjusted mean difference of 1.09° (95% CI: 0.48°–1.71°; *p* = 0.001; Cohen's *d* = 0.42), and at 1 year, with an adjusted mean difference of 1.28° (95% CI: 0.71°–1.85°; *p* < 0.001; Cohen's *d* = 0.54) (Table 2). CS constructs were associated with greater lateral patellar tilt than PS constructs at 2 months, with an adjusted mean difference of 1.02° (95% CI: 0.40°–1.63°; *p* = 0.001; Cohen's *d* = 0.39), and at 1 year, with an adjusted mean difference of 0.82° (95% CI: 0.25°–1.40°; *p* = 0.005; Cohen's *d* = 0.35) (Table 3). No significant approach × construct interaction was identified at 2 months (interaction effect, 0.03°; 95% CI: −1.20° to 1.26°; *p* = 0.966) or at 1 year (− 0.09°; 95% CI: −1.24° to 1.05°; *p* = 0.870).

**Table 2:** Radiological patellar parameters according to surgical approach, with approach × implant interaction *p*‐values.

Parameter mean ± SD	MSV (*n* = 197)	MPP (*n* = 123)	*p*‐Value	Interaction *p*
Patellar tilt 2 months (°)	8.37 ± 2.82	7.27 ± 2.28	<0.001*	0.967
Patellar tilt 1 year (°)	8.31 ± 2.62	7.02 ± 1.98	<0.001*	0.869
Patellar thickness (mm)	24.6 ± 2.9	23.6 ± 2.5	0.002*	0.421
Modified Blackburne–Peel index 2 months	0.77 ± 0.13	0.76 ± 0.15	0.604	0.547
Modified Blackburne–Peel index 1 year	0.76 ± 0.12	0.74 ± 0.15	0.107	0.829
Centred patellar position 2 months (%)	88.4%	85.0%	0.483	—
Centred patellar position 1 year (%)	87.5%	82.9%	0.347	—

Abbreviations: Interaction *p*: surgical approach × insert constraint interaction term; MPP, medial parapatellar; MSV, mini‐subvastus; NS, not significant; —, not applicable (categorical outcome); SD, standard deviation.

* Statistically significant difference *p* < 0.05.

**Table 3:** Radiological and alignment parameters by implant type (CS vs. PS).

Parameter—Mean ± SD	CS (*n* = 209)	PS (*n* = 111)	*p*‐Value
Femoral varus–valgus (°)	1.00 ± 1.71	1.10 ± 1.65	0.274
Tibial varus–valgus (°)	−3.37 ± 1.49	−3.12 ± 1.51	0.307
Femoral rotation PCA (°)	2.69 ± 1.59	2.82 ± 1.78	0.379
Patellar thickness (mm)	24.2 ± 2.7	24.1 ± 2.6	0.791
Modified Blackburne–Peel index 2 months	0.77 ± 0.13	0.76 ± 0.13	0.292
Modified Blackburne–Peel index 1 year	0.76 ± 0.14	0.75 ± 0.13	0.507
Patellar tilt 2 months (°)	8.48 ± 2.78	7.39 ± 2.24	0.001*
Patellar tilt 1 year (°)	8.24 ± 2.48	7.34 ± 2.34	0.005*

Abbreviations: CS, condylar‐stabilised; NS, not significant; PCA, posterior condylar axis; PS, posterior‐stabilised; SD, standard deviation.

* Statistically significant difference *p* < 0.05.

### Secondary radiographic outcomes

Patellar thickness was slightly greater in the MSV group than in the MPP group (24.6 ± 2.9 vs. 23.6 ± 2.5 mm, *p* = 0.002), but did not differ according to insert constraint (24.2 ± 2.7 vs. 24.1 ± 2.6 mm, *p* = 0.791) (Tables 2 and 3). The modified Blackburne–Peel index did not differ between surgical approaches or insert constraints at either time point. The proportion of centred patellae did not differ significantly between MSV and MPP at 2 months (88.4% vs. 85.0%, *p* = 0.483) or 1 year (87.5% vs. 82.9%, *p* = 0.347). Qualitative patellar centreing showed small exploratory differences according to insert constraint and should be interpreted cautiously.

## DISCUSSION

The principal observation of this study was that postoperative static patellar tilt differed by approximately 1° across surgeon‐dependent surgical approach groups and between composite CS and PS construct groups, without a significant approach × construct interaction. These findings represent descriptive group‐level radiographic differences and do not isolate the independent effects of surgical approach or insert constraint.

Robotic‐assisted TKA has been shown to improve the accuracy and reproducibility of component positioning compared with conventional instrumentation [7, 12]. This is particularly relevant for the patellofemoral joint, because femoral rotational malposition has been associated with altered patellar tracking and increased patellofemoral contact pressures [15]. Beyond coronal and rotational accuracy, robotic platforms allow intraoperative adjustment before final bone cuts, integrating ligament balance, component position and patellofemoral behaviour [1, 23]. No statistically significant between‐group differences were detected in the recorded final femoral and tibial coronal alignment or femoral rotation parameters. The study, therefore, describes only the small residual static radiographic differences observed within this specific robotic‐assisted cohort.

The effect of surgical approach on patellofemoral mechanics remains controversial. The MSV approach preserves the quadriceps tendon and vastus medialis continuity, and has been associated with faster early recovery in some studies [2, 4, 21]. Conversely, the MPP approach provides reliable exposure but involves proximal extension through the quadriceps tendon [2]. Previous studies comparing minimally invasive or quadriceps‐sparing approaches with parapatellar or midvastus approaches have reported variable effects on patellar tracking and anterior knee pain [11, 17, 18]. In the present study, MSV was associated with statistically greater lateral patellar tilt than MPP at both follow‐up assessments. This finding contrasts with the theoretical advantage expected from preservation of the extensor mechanism. Potential explanations include differences in patellar mobilisation or eversion, intraoperative extensor‐mechanism tension, soft‐tissue handling, patient selection and surgeon‐related technical factors. However, these variables were not directly assessed. Furthermore, the approach was structurally confounded by surgeon preference. The mechanism underlying this association, therefore, cannot be determined from the present data, and its clinical significance remains unknown.

Insert constraint was also associated with small differences in patellar tilt. CS implants showed slightly greater tilt than PS implants, despite both femoral components shared the same anterior flange and trochlear geometry, the constructs differed in insert design, posterior cruciate ligament management and flexion‐balance targets. Patellofemoral complications have previously been linked to prosthetic design, particularly trochlear morphology and the interaction between femoral component shape and patellar tracking [14, 15]. In this study, however, the difference cannot readily be attributed to trochlear design. Differences in posterior cruciate ligament management, flexion balance or sagittal‐plane femorotibial kinematics could theoretically influence patellofemoral behaviour. However, these potential mechanisms were not directly evaluated in the present study. Moreover, construct selection was not randomised and depended on surgeon preference and institutional practice. Residual confounding and construct‐selection bias, therefore, remain plausible explanations for the observed association.

Patellar height, assessed using the modified Blackburne–Peel index, did not differ between approaches or insert constraints. This is reassuring because patellar height can influence extensor mechanism mechanics and may affect patellofemoral function after TKA [3, 24]. Patellar thickness was slightly greater in the MSV group; because the patella was not resurfaced, this likely reflects baseline morphology rather than a procedural effect. Qualitative patellar centreing showed small exploratory differences, but this parameter was assessed categorically rather than as a continuous displacement measurement and should not be overinterpreted.

The clinical relevance of the observed differences remains uncertain. Some authors have reported associations between patellar tilt and functional outcomes after TKA [6], whereas others found that small variations in patellar tilt did not meaningfully affect PROMs after modern TKA [16]. Di Quattro et al. similarly reported that patellofemoral alignment safe zones in robotic‐assisted TKA did not clearly translate into improved short‐term outcomes, although they influenced resurfacing decisions [9]. In the present study, no PROMs, anterior knee pain scores or dynamic tracking assessments were available. Accordingly, the approximately 1° differences observed should be interpreted as radiographic associations, and their clinical significance remains unknown.

This study has several limitations. First, 340 of the 669 screened TKAs were excluded because the patella had been resurfaced, a decision based partly on anterior knee pain, patellofemoral osteoarthritis and surgeon preference. Differential selection among the surgeon‐dependent approach groups cannot be excluded, and the findings apply only to this selected population of nonresurfaced TKAs. Second, preoperative patellar tilt, translation, morphology and patellofemoral osteoarthritis severity were not systematically available. Given that the observed postoperative differences were approximately 1°, even a small baseline imbalance could account for some or all of these findings. Third, preoperative patellar tilt, patellar translation, patellar morphology and patellofemoral osteoarthritis severity were not systematically available. Consequently, postoperative patellar alignment could not be interpreted in relation to each patient's baseline patellofemoral anatomy, and residual confounding related to preoperative patellofemoral characteristics cannot be excluded. Fourth, tibial component rotation, which may influence patellar tracking, was not routinely exported from the robotic platform and therefore could not be analysed. It therefore represents a potentially important unmeasured confounder. In addition, because a posterior tibial slope target of 3° was applied in all cases, the influence of variations in tibial slope on patellar alignment could not be assessed. Fifth, the surgical approach was determined by surgeon preference, with two surgeons routinely performing the mini‐subvastus approach and one surgeon the medial parapatellar approach. Consequently, surgeon and approach were inherently confounded, precluding attribution of the observed differences solely to the surgical approach. Sixth, the present study was designed to compare the effects of surgical approach and insert constraint within a standardised robotic‐assisted functional alignment strategy rather than to identify independent radiographic predictors of patellar tilt. Therefore, adjusted regression analyses exploring the association between individual robotic alignment parameters and patellar tilt were beyond the scope of this study. Finally, although good‐to‐excellent intraobserver reliability was demonstrated, interobserver reliability was not assessed. Consequently, reproducibility across different observers remains unknown.

Future studies should combine robotic planning data, dynamic patellar assessment and patient‐reported outcomes to determine whether these small radiographic variations translate into measurable clinical effects.

## CONCLUSION

A robotic‐assisted functional positioning strategy provided consistent static patellofemoral alignment across different surgical approaches and insert constraints. Although statistically significant differences in patellar tilt of approximately 1° were observed, no significant interaction was found between surgical approach and insert constraint.

## AUTHOR CONTRIBUTIONS

*Study design, manuscript writing*: Clément Favroul. *Manuscript writing, data collection, statistical analysis*: Mehdi Drissi Kaitouni. *Manuscript editing*: Elvire Servien and Cécile Batailler. *Study design, supervision, literature review and manuscript editing*: Sébastien Lustig. All authors read and approved the final manuscript.

## CONFLICT OF INTEREST STATEMENT

Clément Favroul and Mehdi Drissi Kaitouni declare that he have no conflict of interest. ES: consultant for Corin. Cécile Batailler: consultant for Stryker, Smith & Nephew. Sébastien Lustig: consultant for Stryker, Smith & Nephew, Heraeus, Depuy Synthes; Institutional research support from Groupe Lepine, Amplitude; Editorial Board for Journal of Bone and Joint Surgery (Am).

## ETHICS STATEMENT

All procedures were performed in accordance with the ethical standards of the institutional and/or national research committee, the 1964 Helsinki Declaration and its later amendments or comparable ethical standards. Data collection and analysis were carried out in accordance with MR004 Reference Methodology from the Commission Nationale de l'Informatique et des Libertés (Ref. 2229975V0) obtained on 6 May 2023. The study was registered and filed on the Health Data Hub website. As per institutional standards, formal patient consent is not required for this type of study.

## Data Availability

Data available on request due to privacy/ethical restrictions.
